# The inflammasomes, immune guardians at defence barriers

**DOI:** 10.1111/imm.12989

**Published:** 2018-09-06

**Authors:** Pablo Palazon‐Riquelme, Gloria Lopez‐Castejon

**Affiliations:** ^1^ International Centre for Infectiology Research INSERM U1111 CNRS UMR5308 École Normale Supérieure de Lyon Claude Bernard Lyon 1 University Lyon France; ^2^ Manchester Collaborative Centre of Inflammation Research The University of Manchester Manchester UK; ^3^ The Lydia Becker Institute of Immunology and Inflammation Faculty of Biology, Medicine and Health Manchester Academic Health Sciences Centre University of Manchester Manchester UK

**Keywords:** epithelial cell, inflammasome, inflammation

## Abstract

As a result of its strategic location, the epithelium is constantly exposed to a wide variety of pathogen and danger signals. Traditionally, the epithelium has been perceived as a defensive but passive barrier; however, it has now become evident that the epithelium senses and actively responds to these signals in order to maintain barrier homeostasis and contributes to the inflammatory response. One way it does this is by producing pro‐inflammatory cytokines including interleukin‐1*β* (IL‐1*β*) and IL‐18. These two cytokines are synthesized as inactive precursors, the maturation of which is mediated by pro‐inflammatory caspases after the activation and assembly of macromolecular complexes called inflammasomes. Epithelial cells express a large panel of inflammasome components, and although the molecular mechanisms underlying the activation of these complexes in haematopoietic cells are well understood, how epithelial cells react to danger signals to activate the inflammasome remains unclear. We review and discuss how different inflammasomes contribute to barrier homeostasis and inflammation at several barrier sites, their mechanisms and how their aberrant regulation contributes to disease at the different epithelia.

## Introduction

As a result of its essential role as a physical barrier between our body and the external environment, epithelial cells are some of the most specialized cells of the human body. The epithelium is often targeted by a myriad of pathogens, including bacteria, fungi and viruses, but also by particulate matter such as tobacco smoke and air pollution.^1^ When it comes to respond to these threats, resident macrophages play an essential role.^2^ Macrophages express numerous membrane and cytosolic receptors to detect threats, and the study of those (including the activation of inflammasome) has been widely studied. However, in recent years and breaking the paradigm of epithelial cells as a passive barrier, epithelial cells have also been shown to play an important and active role in these immune responses.

Epithelial cells can sense threats through membrane‐bound receptors such as Toll‐like receptors (TLRs) TLR4 and TLR5,^3^ but also through intracellular receptors such as TLR7, TLR9, absent in melanoma 2 (AIM2; a receptor that recognizes cytosolic foreign DNA) and nucleotide oligomerization‐domain‐like receptors (NLRs) recognizing sterile and pathogen‐associated signals.^4^, ^5^ Upon sensing damage‐ and pathogen‐associated molecular patterns (DAMPs and PAMPs), the epithelium secretes a plethora of immune mediators, constituting the first layer of defence that alerts the immune system to the presence of a threat. Among these immune mediators, there are mucins, defensins and lysozymes; cytokines such as granulocyte–macrophage colony‐stimulating factor, tumour necrosis factor‐*α*, interleukin‐6 (IL‐6) and also the two unique cytokines IL‐1*β* and IL‐18.^6^ Unlike other cytokines, IL‐1*β* and IL‐18 are synthesized as pro‐forms that need to be cleaved by caspase‐1 after the activation of a multiprotein complex called inflammasome.

## The inflammasome: a well‐regulated immune platform also present in non‐myeloid cells

**Figure 1:**
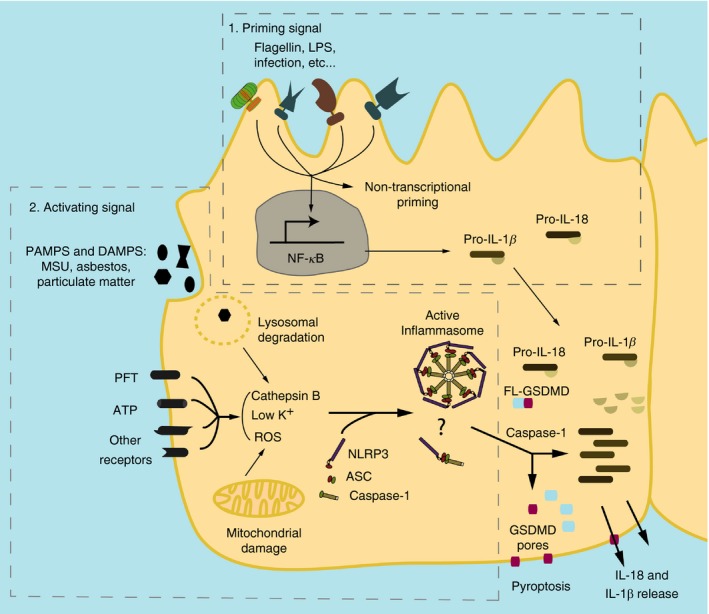
Canonical activation of NACHT, LRR and PYD domains‐containing protein 3 (NLRP3) inflammasome. Inflammasome needs a priming signal^1^ such as lipopolysaccharide (LPS) that triggers the nuclear factor‐*κ*B (NF‐*κ*B) pathway leading to an increase in the production of pro‐interleukin‐1*β* (pro‐IL‐1*β*). The priming signal also has a non‐transcriptional function, deubiquitinating NLRP3.^12^ A second signal, named activation signal,^2^ is triggered after the recognition of pathogen‐associated molecular patterns (PAMPs) and damage‐associated molecular patterns (DAMPs) through specific receptors, membrane disruption or lysosomal uptake. This activating signal will trigger a disruption of cell homeostasis. This disruption will be sensed in different ways, including the release of cathepsin B by damaged lysosomes, increased production of mitochondrial reactive oxygen species (ROS) and/or potassium efflux. This potassium efflux will be detected by cytosolic receptors such as NLRP3. The recognition of the signal will foster NLRP3 oligomerization and the formation of the active complex of the inflammasome after recruitment of apoptosis‐associated speck‐like protein containing a caspase activation and recruitment domain (ASC) and caspase‐1. The active inflammasome will cleave procaspase‐1 generating active caspase‐1, which will lead to the production and release of the pro‐inflammatory cytokines IL‐1*β* and IL‐18. Caspase‐1 will also cleave full‐length gasdermin‐D (FL‐GSDMD), which will create pores in the membrane. These pores are suggested to be one mechanism of release for IL‐18 and IL‐1*β* but not the only one. The gasdermin‐D pore will also produce a form of pro‐inflammatory cell death known as pyroptosis. **?**, it is unclear whether epithelial cells can form inflammasome multimeric specks or present only filament oligomerization; MSU, monosodium urate; PFT, pore‐forming toxins.

The canonical assembly of the inflammasome is a well‐regulated process that starts after the recognition of PAMPs or DAMPs by cytosolic receptors. The cytosolic receptor acts as a sensor protein and typically belongs to the NLR family but the activation of other receptors such as AIM2 also leads to the formation of an inflammasome^5^ (Table 1). The most studied inflammasome is NLRP3 because of its ability to respond not only to pathogens but also to sterile stimuli.^7^ Activation of the cytosolic receptor leads to the recruitment of the effector enzyme capase‐1. Depending on which cytosolic receptor is activated, the recruitment of the effector enzyme, caspase‐1, will require an adaptor molecule known as apoptosis‐associated speck‐like protein containing a caspase activation and recruitment domain (ASC) or not (Table 1). Upon oligomerization of the inflammasome, the effector enzyme caspase‐1 is activated and leads to the cleavage, maturation and release of IL‐1*β* and IL‐18, and to a form of cell death known as pyroptosis^8^ (Fig. 1). It is important to mention that, although most of the inflammasome‐forming receptors need ASC for their function, NLRP1b and NLRC4 have been shown to have both ASC‐dependent and ASC‐independent functions. Although ASC is dispensable (but significantly increases) NLRP1b function, NLRC4‐mediated pyroptosis (but not cytokine release) is ASC‐independent.^9^, ^10^, ^11^

**Table 1:** Main inflammasome‐forming receptors in epithelial cells

Subfamily	Receptor	Requirement for ASC	Main activating signal	Main epithelia involved
NLRP	NLRP1	Yes^a^	Anthrax toxin and ATP	Oral, airway^22^, ^23^, ^24^
	NLRP3	Yes	Ionophores, crystals, ATP, bacterial toxins	Oral, airway, intestinal, skin^22^, ^23^, ^25^, ^26^
	NLRP6^b^	Yes	Microbiota‐modulated metabolites (taurine)	Intestinal^27^
	NLRP9b	Yes	Rotavirus dsRNA	Intestinal^28^
NLRC	NLRC4/IPAF	Yes^a^	Flagellin (in mouse)/Type III secretion system (in human)	Intestinal^29^
Other inflammasome‐forming sensors	AIM2	Yes	dsDNA	Oral, skin^23^, ^30^

Not all the inflammasome‐forming receptor proteins require the adaptor protein apoptosis‐associated speck‐like protein containing a caspase activation and recruitment domain (ASC) to activate caspases‐1, however, when present, ASC potentiates inflammasome activity. Also shown the main activating signals and epithelial distribution.

a Both nucleotide NACHT, LRR and PYD domains‐containing protein 1 (NLRP1) and NLRC4 protein have a caspase recruitment domain and have shown activity in the presence or absence of ASC.^9^, ^31^

b The ability of NLRP6 to form inflammasomes has only been suggested but not directly shown.

Although canonical inflammasomes rely on the activation of caspase‐1, cells can also form ‘non‐canonical’ inflammasomes, which lead to the activation of caspase‐11 (caspase‐4 and caspase‐5 in humans) or caspase‐8.^13^, ^14^ Caspase‐11 acts as an intracellular lipopolysaccharide (LPS) sensor by direct binding using its caspase activation and recruitment domain (CARD). Interestingly, this process is widely extended into myeloid and non‐myeloid cells.^15^ Therefore, caspase‐11 could be a widespread mechanism to detect intracellular Gram‐negative bacteria in cells, including infected epithelial cells. Upon LPS recognition, caspase‐11 is activated and causes K^+^ efflux, which induces NLRP3 canonical inflammasome formation and IL‐1*β* and IL‐18 release.^16^

Both IL‐1*β* and IL‐18 are potent pro‐inflammatory cytokines with a pivotal role during the first steps of inflammation and their deregulation is extremely detrimental to health. Hence, these are tightly regulated proteins, not only at transcriptional level, but also post‐transcriptionally by their activation within the inflammasome. Although IL‐1*β* and IL‐18 are activated by the inflammasome in a similar manner, their contribution to the inflammatory response is very different. Interleukin‐1*β* drives inflammation by controlling the recruitment of neutrophils to the site of infection, the induction of IL‐8 by epithelial cells and the release of IL‐17 from T cells.^17^ On the other hand, the main role of IL‐18 is to foster the activation of natural killer and T cells and the release of interferon‐*γ*.^18^, ^19^, ^20^ Although most studies focus on the release of IL‐1*β* and IL‐18 by immune cells such as macrophages, epithelial cells are also able to release these pro‐inflammatory cytokines.^19^, ^21^ However, the mechanistic insights by which this occurs remain unclear.

**Figure 2:**
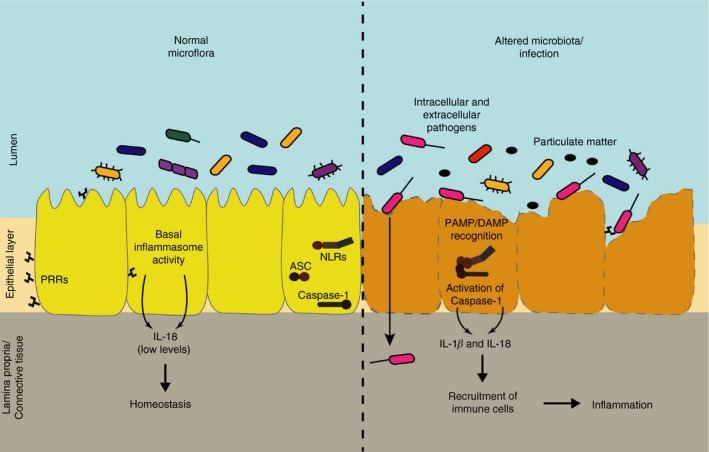
During healthy conditions epithelial cells do not respond (or have a weak response) to commensal bacteria or non‐invasive microorganisms. However, epithelial cells still release basal levels of interleukin‐18 (IL‐18).^19^, ^45^, ^46^ The lack of response from epithelial cells to commensal bacteria is partially due to differential distribution of pattern recognition receptors (PRRs) in the membrane, predicted to foster recognition of only invasive pathogens. After invasion and disruption of the epithelial layer by bacterial pathogens, viruses or exposure to sterile agents, epithelial cells recognize pathogen‐ or danger‐associated molecular patterns (PAMPS and DAMPS) and activate inflammasome. Inflammasome activation in epithelial cells leads to the release of IL‐1*β* and IL‐18, recruiting immune cells to the site of infection and inflammation.

As a result of their essential role as triggers of innate immunity, and in order to tightly control their release, IL‐1*β* and IL‐18 are synthesized as pro‐forms lacking a signal peptide. The maturation and release of these pro‐inflammatory cytokines are regulated by the assembly of a multiprotein complex known as the inflammasome, also present in epithelial cells (Table 1; Fig. 2).^22^

Despite caspase‐1 being the main caspase involved in this process, others such as caspase‐4/5 (human orthologues of murine caspase‐11) and caspase‐8 can also play an important role.^13^, ^32^ Recently, a new protein‐mediating pyroptosis has been described: gasdermin‐D.^33^, ^34^ Gasdermin‐D is activated by caspase‐1 and forms pores in the membrane, which leads to cell death.^35^, ^36^, ^37^, ^38^ It has been recently proposed that IL‐1*β* and IL‐18 release happens through gasdermin‐D pores;^39^ even so, further research is needed. Gasdermin‐D is also expressed in epithelial cells, and its involvement on inflammasome activity has been reported. Although no direct cleavage of gasdermin‐D has been shown in epithelial cells, the pore‐forming protein has been shown to be necessary for NLRC4‐mediated and NLRP9b‐mediated pyroptosis.^28^, ^40^, ^41^ This would show that gasdermin‐D is functional and important for epithelial cell inflammasome function. Interestingly, in recent years, it has been reported that under certain circumstances the release of pro‐inflammatory cytokines and cell death are uncoupled upon inflammasome activation in several cell types such as neutrophils, monocytes and macrophages.^39^, ^42^, ^43^ This is particularly important for epithelial cells where the conservation of the integrity of the epithelial layer is key to maintain barrier homeostasis^44^ (Fig. 2). Whether the release of IL‐1*β* and IL‐18 is uncoupled from cell death in epithelial cells remains unknown.

Despite increasing research in this area, there are still unanswered questions regarding inflammasome activation in the epithelium (Fig. 2). The epithelium is exposed to mechanical and chemical damage associated with the exterior milieu as well as to a wide range of microorganisms colonizing our body. Hence, the inflammasome activity has to be tightly regulated in epithelial cells, not only to prevent an inflammasome response to healthy microbiota but also to potentiate the response to an altered microbiome. In this review, we will summarize the current knowledge on inflammasome activation at different barrier sites, and how these molecular complexes contribute not only to maintenance of barrier homeostasis but also to disease.

### Gut epithelial cells

The most studied inflammasome‐forming epithelium is the gut epithelium. In this barrier, the heavy colonization of commensal microbiota makes the tight regulation of the inflammatory cascade crucial. How gut epithelial cells respond to invasive pathogens and not to commensal bacteria remains only partially understood. It has been shown that differential distribution of TLRs on the membrane of gut epithelial cells allows the response to invasive microorganisms not just after apical contact but after the disruption of the epithelium^47^ (Fig. 2). The requirement of invasion or damage of epithelium integrity to activate the immune function of epithelial cells guarantees that repair mechanisms and inflammatory processes are triggered only when needed. In addition to TLRs, the cytosolic localization of the inflammasome‐forming sensors NLRs and AIM2, and their ability to respond to damage signals as well as to pathogens, places them as ideal platforms to detect pathogen invasion.

The role of inflammasomes at this barrier became apparent after early reports showed that intestinal epithelial cells (IEC) were able to up‐regulate IL‐1*β* in response to infection^48^ as well as being the principal contributors to the total levels of IL‐18 in the gut under physiological conditions.^49^ Since then, an increasing amount of evidence has shown an involvement of the inflammasome in maintaining homeostasis in this barrier as well as responding to pathogens.

The gut epithelium has been shown to rely on the NLRC4 inflammasome to respond to *Citrobacter rodentium*, a mouse model of enteropathogenic *Escherichia coli* infection.^29^, ^50^ Interestingly, epithelial NLRP3 is also involved in *C. rodentium* clearance as *Nlrp3* ^−/−^ mice show higher bacterial burden and higher epithelium invasion.^51^ This effect is likely to be mediated by both IL‐18 and IL‐1*β* as *Il‐18* ^−/−^ and *Il‐1β* ^*−/−*^ mice present a similar phenotype to the *Nlrp3* ^−/−^ mouse when challenged with *C. rodentium*.^50^ These findings have also been confirmed in human cells after *E. coli* infection of the epithelial cell line Caco‐2 induced increased levels of IL‐18 release.^52^ Apart from this role in infection, caspase‐1 and NLRC4 have also been reported as essential in preventing colonic inflammation‐induced tumorigenesis by regulating the response of epithelial cells to injury, highlighting the role of NLRC4 in epithelial cells.^53^, ^54^

In another model of infection, inflammasome‐dependent release of IL‐18 has been shown to mediate natural killer cell recruitment after *Salmonella typhimurium* infection.^55^ Although authors do not investigate the origin of gut IL‐18 in this paper, the above‐mentioned studies suggest that this IL‐18 is likely to be of epithelial cell origin. Using the same infection model, it has been shown that inflammasome‐forming sensors are also involved in the extrusion and expulsion of intestinal epithelial cells. During *S. typhimurium* infection*,* caspase‐4 mediates epithelial cell expulsion through a process dependent on pyroptosis.^56^ This process is epithelial cell intrinsic and independent of cytokine signalling, showing a specific role for pyroptosis and for non‐canonical inflammasomes.^57^ However, this is not the only inflammasome‐dependent mechanisms for epithelial cell expulsion. In another study exploring the role of NLRC4 in gut epithelial cells, authors showed that caspase‐1 and gasdermin‐D are dispensable for IEC expulsion from the epithelial layer upon treatment with the NLRC4 activator FlaTox (*Legionella pneumophila* flagellin fused to *Bacillus anthracis* lethal factor), as cells were still expelled from the epithelium in the absence of these mediators. In contrast, the expelled cells were not dead, implying that both caspase‐1 and gasdermin‐D are required for IEC pyroptosis upon NLRC4 activation.^41^ How the two mechanisms can act together is still not understood. Although this is an important mechanism necessary for the maintenance of normal microbiota and pathogen clearance, all these studies highlight the danger of excessive inflammasome response and gasdermin‐D‐mediated pyroptosis in gut epithelial cells. An excessive activation of pyroptosis could eventually lead to loss of membrane integrity and systemic infection.

Another NLR involved in the relationship between gut epithelium and bacteria is NLRP6. Recent findings emphasize the importance of having proper controls to elucidate the role of different inflammasomes in immunity, especially in maintaining commensal bacteria. Although the role of NLRP6 in gut epithelial cells is still not totally clear, NLRP6 is highly expressed and functional in gut epithelial cells.^27^ Mamantopoulos *et al*.,^58^ in two different animal facilities, dismissed a role of NLRP6 in maintaining homeostasis in the gut microbiota at steady state. However, it has been proposed that NLRP6 does contribute to microbiota balance, but this contribution is dependent on microbiota community structure (and especially in the presence of pathobionts such as *Helicobacter* spp.). Both of these studies point to familial transmission of microbiota rather than NLRP6 impairment for the observed changes in previous studies.^59^, ^60^ How the cells activate NLRP6 and whether NLRP6 forms an active inflammasome are still not fully answered questions. No direct evidence shows NLRP6 forming an inflammasome but *Nlrp6* ^*−/*−^ studies mimic the results of *Caspase‐1* ^*−/−*^ and *Asc* ^*−/−*^, suggesting that this might be the case.^61^, ^62^ Recently, taurine, an amino acid present in bile acid and shown to regulate gut microbiota, has been proposed to be the activating signal for NLRP6 as it induces IL‐18 secretion in wild‐type mice but not in *Nlrp6* ^*−/−*^ mice.^62^, ^63^ Although this finding needs to be further tested and investigated, it would be highly interesting as the gut microbiota in return has also been shown to regulate bile acid metabolism and especially taurine.^64^ Further research is needed to elucidate the molecular events leading to NLRP6 activation.

A highly prevalent condition of the gut is inflammatory bowel disease (IBD, including Crohn's disease and ulcerative colitis). IBD is a chronic inflammation of the digestive system associated with the alterations of the gut microbiota.^65^ The most common mouse model of IBD is to induce colitis using dextran sodium sulphate (DSS), which damages the epithelium, increasing gut permeability.^66^ Inflammasome responses in experimental models of colitis are well characterized, but mainly in monocytes and macrophages.^67^ In such models, NLRP3 inhibition ameliorates DSS‐induced colitis.^68^, ^69^, ^70^ However, these studies fail to show a specific role for epithelial cells, despite these being the main cell type affected by IBD. In fact, recent studies suggest that NLRP3 is not expressed by epithelial cells in the colon of mice, placing tissue‐resident macrophages as the principal contributors to this NLRP3‐dependent response.^71^ These studies suggest that although NLRP3 is a main actor in IBD, the epithelial cell layer's contribution to IBD might be NLRP3 independent. Surprisingly, epithelial cell‐specific depletion of capase‐1 induces decreased IL‐18 levels and decreased pathology in a DSS‐induced colitis model, pointing to the involvement of another inflammasome‐forming receptor.^72^ This is consistent with the finding that during colitis, IL‐18 produced by epithelial cells exacerbates pathology. Interestingly, epithelial IL‐18 signalling during colitis targets epithelial cells themselves as *Il‐18R* ^*−/−*^ in haematopoietic cells fails to rescue mice from colitis, whereas specific *Il‐18R* ^*−/−*^ in epithelial cells protects mice from DSS‐induced colitis.^73^ Recent studies also point to a role of AIM2 in DSS‐induced colitis. AIM2 knockout mice are more severely affected by colitis and present a higher bacterial burden; however, how AIM2 is activated or how DNA is delivered in the epithelial cells during colitis is unknown. In fact, authors suggest that the role of AIM2 in colitis is to control the anti‐microbial peptide production (such as *α*‐ and *β*‐defensins) in IEC, and to regulate tissue repair, activating an IL‐18‐dependent pathway.^74^, ^75^

All these reports together show an increasingly recognized role of inflammasomes in innate immunity driven by gut epithelial cells and places the inflammasome response as a coordinated process with several outcomes. However, a more profound study using IEC or myeloid‐cell‐specific knockout mice would be useful to better understand the role of epithelial cells in barrier defence.

### Lung epithelium

Due to the extended area of contact between internal and external stimuli, airway epithelium is a primary target for pathogens and damaging agents. Lung epithelial cells have been shown to activate the inflammasome and release IL‐1*β* and IL‐18 upon a diverse range of stimuli such as influenza virus, ozone, particulate matter, or asbestos.^76^, ^77^, ^78^, ^79^ However, despite the importance of the release of IL‐1*β* and IL‐18 in this organ, the events leading to their release from epithelial cells are still not well understood and are the subject of controversy, probably partly due to different cell types used in different studies. For instance, the involvement of caspase‐1 is not clear because some studies show that epithelial cell lines (Beas2Bs, 16HBE, Calu‐3) and also primary cells (human bronchial epithelial cells) express little to no caspase‐1 in an infection model, whereas others show a role for caspase‐1 in epithelial cells (for instance in Beas2Bs, mouse tracheobronchial epithelial cells and A549).^80^, ^81^, ^82^, ^83^ It has been suggested that as some of these cells do not express the inflammasome component, caspase‐11 is responsible for the inflammasome response to infections, probably by direct biding of LPS.^84^ These differential results highlight the importance of analysing the expression of inflammasome components in the lung epithelial cell lines before conducting inflammasome experiments and the need to work in mouse and human primary cells.

As in the gut, the lungs constitute an area that is heavily colonized by commensal bacteria. However, in contrast to the gut, the lung epithelium is also heavily exposed to agents causing sterile inflammation such as particulate matter present in tobacco smoke or urban pollution.

Despite the fact that airway macrophages respond strongly to particulate matter due to their increased ability to phagocytose, epithelial cells also respond to air pollutants.^85^ Human bronchial epithelial cells (Beas2B) have been shown to up‐regulate, process and release IL‐1*β* in response to crystal silica exposure, which is found in the air and taken up by epithelial cells.^83^ Interestingly, in the same study, they also detect release of High mobility group box 1 protein (HMGB1) from epithelial cells after silica exposure. HMGB1 is a potent danger signal also released by A549 cells under infection.^82^ These observations are relevant because the crosstalk among epithelial cells and between epithelial cells and immune cells is important for tissue repair. Other urban particulate matter (particulate matter < 10 µm) can also activate the inflammasome in an NLRP3‐dependent manner.^79^ This could be particularly important in urban or industrial areas. This particulate matter uptake by epithelial cells and inflammasome activation could worsen already existing conditions or cause new ones. In fact, it has been suggested that following periods of poor air quality (characterized by higher levels of particulate matter), inflammasome activity could be contributing to the exaggerated response to viruses such as influenza and allergy.^21^, ^86^

Another major agent for lung dysfunction, cigarette smoke, increases the release of IL‐1*β* in human lung epithelial cells, and this increase is even higher in patients with chronic obstructive pulmonary disease (COPD).^87^ Indeed, IL‐1*β* has been shown to be a marker of COPD severity as individuals with COPD present higher levels of IL‐1*β* in serum.^88^ In contrast, another study shows that cigarette smoke reduces the inflammatory response in mice treated with another NLRP3‐activating crystal: asbestos.^89^ Although contradictory, the reduction of the inflammatory response could be explained by the promotion of the proteasomal degradation of NLRP3 mediated by its ubiquitination.^90^

In addition to sterile agents, the lung is also exposed to pathogens. A condition where the lung epithelium integrity is compromised is during aspergillosis. Being inhaled every day, the spores of *Aspergillus fumigatus* invade the lung of not only immunocompromised patients but also patients with COPD.^91^ Inflammasomes have long been involved with the innate immune response to *A. fumigatus*, and recent studies show that inflammasome activation occurs early during infection and is key for macrophage anti‐fungal activity.^92^, ^93^ Interestingly, *β*‐glucan, a component of the *A. fumigatus* cell wall, upon dectin‐1‐binding activates NLRP3 inflammasome in bronchoepithelial cells.^94^ In fact, Syk tyrosine kinase, a kinase activated through dectin‐1, has been shown to be involved in inflammasome activation by *A. fumigatus* in monocytes, reinforcing the role of *β*‐glucan in inflammasome activation in lung epithelial cells.^95^ Both studies point at reactive oxygen species, a major mediator of inflammasome activation, as a key factor during aspergillosis.

The complexity of the lung together with the distinct environments and stimuli that lung epithelial cells face makes the study of the epithelial inflammasome in this organ quite challenging. However, these studies show the inflammasome in the lung as a clear therapeutic target to ameliorate a broad range of pathologies with an inflammatory component.

### Oral epithelium

An entry gate for nutrients, the oral mucosa is a heavily exposed environment where particulate matter, and bacterial and fungal agents constantly challenge the homeostasis of the epithelium.

Gingivitis is inflammation of the gums, where the gum pulls away from the bone, creating cavities that are colonized by pathogens, which if left untreated can lead to periodontitis. One of the main pathogens colonizing these cavities and causing the disease is *Porphyromonas gingivalis*. Periodontitis has been linked to increased expression of inflammasome‐sensing proteins NLRP3 and AIM2, mainly in the epithelial layer.^22^, ^96^ Moreover, a polymorphism in other NLR family member, NLRC5, has been linked to increased susceptibility to periodontitis,^97^ although how it contributes to this pathology has not been investigated. NLRC5 has a caspase recruitment domain but it has not been shown to form inflammasomes on its own. In fact, NLRC5 can associate with NLRP3 in response to different PAMPs and DAMPs to cooperatively activate the inflammasome.^98^ This would suggest that the polymorphisms of NLRC5 induced to increased susceptibility to periodontitis are the consequence of an aberrant assistance to NLRP3 to form inflammasomes. Other roles for NLRC5 have been described including negative regulation of nuclear factor‐*κ*B,^99^ interferon signalling^99^, ^100^ and regulation of major histocompatibility complex class I genes,^101^ all of which could also explain its contribution to periodontitis.

As expected, infection of gingival epithelial cells with *P. gingivalis* induced an increase in *Il‐1β* gene expression. However, IL‐1*β* was left unprocessed and accumulated intracellularly. Interleukin‐1*β* was only processed and released after the activation of the ATP‐gated receptor P2X7, which led to NLRP3 inflammasome formation. The P2X7R is also involved in NLRP3 inflammasome formation and release of mature IL‐1*β* from salivary epithelial cells, as the release of this pro‐inflammatory cytokine is dampened in P2X7R knockout mice.^102^, ^103^ Why IL‐1*β* remains uncleaved and is intracellular during *P. gingivalis* infection remains an interesting question. We could speculate that the immune machinery of epithelial cells might reduce the activation of caspase‐1 during chronic infection. This mechanism could potentially dampen immune reactions to commensal bacteria and prevent pyroptosis to maintain the integrity of the epithelial layer. In fact, in macrophages, it has been shown that IL‐1*β* can be released via exosomes in a mechanism dependent on NLRP3 and ASC but independent of caspase‐1.^104^, ^105^ Potentially, IL‐1*β* produced by epithelial cells could be stored and released on exosomes to have two potential effects. (i) Be processed by other cells (potentially immune cells) to avoid activating caspase‐1 and compromising the epithelial layer in epithelial cells. (ii) Have an effect in areas distant to the site of infection. Although epithelial cells have been shown to release cytokines trapped in exosomes, it is unknown whether IL‐1*β* could follow the same pathway.^106^

Interestingly, another NLR family member, NLRX1, has recently been shown to potentiate the inflammasome response to ATP in gingival epithelial cells through the generation of mitochondrial reactive oxygen species. However, how NLRX1 (not predicted to form inflammasomes) potentiates the response to ATP is not well understood and might assist NLRP3 in its function in a similar manner to NLRC5.^107^

Although inflammasome activity is required for optimal control and clearance of pathogens, over‐activation could lead to unwanted outcomes. Excessive levels of IL‐1*β* and of pyroptosis have been linked to bone loss during periodontitis and to malignancy during oral carcinogenesis.^108^, ^109^ Epithelial cells can avoid this and reduce the extent of inflammasome activity during acute infection through the stress‐induced enzyme haem oxygenase‐1. This enzyme has anti‐oxidant and anti‐inflammatory effects, and can dampen LPS signalling and inflammasome activity in oral epithelial cells, although the exact mechanism by which this occurs is not clear.^110^

### Skin epithelial cells

The skin is the main physical barrier that protects our body from the external environment, therefore it is active in the immune control of pathogen invasion. Immune homeostasis in the skin is essential to maintain skin integrity and its role as a barrier.

Although the role of the inflammasome in skin is not well characterized, mutations in different genes coding for NLR‐forming inflammasomes have been associated with skin conditions. Even before the discovery of the inflammasome, mutations in the *NLRP3* gene were found to cause a spectrum of diseases known as cryopyrin‐associated periodic syndromes (or CAPS), which are associated with skin inflammation and rashes.^111^ Rashes typically disappear, or are controlled, after anti‐IL‐1*β* therapy, showing a role for IL‐1*β* signalling in skin rashes and in CAPS in general.^112^

Recently the first genetic evidence connecting inflammasome signalling to non‐fever skin diseases has been found. Gain of function mutations in the NLRP1 sensor protein cause two overlapping skin disorders: multiple self‐healing palmoplantar carcinoma and familial keratosis lichenoides chronica. These mutations lead to spontaneous inflammasome activation and paracrine IL‐1 signalling in keratinocytes from these patients.^24^ Polymorphisms of *NLRP1* have also been associated with the autoimmune disease vitiligo, a condition characterized by the development of white patches in the skin due to lack of melanin.^113^ This is believed to occur through higher IL‐1*β* production and NLRP1 activation, although the specific mechanism by which the NLRP1 inflammasome shows greater functional activity is not known.^114^

Inflammasome has also been involved in another chronic inflammatory syndrome affecting the skin. Deregulated levels of IL‐1*β* and AIM2 inflammasome activity by keratinocytes have been linked to psoriatic disease. Abnormally high levels of IL‐1*β* could potentiate a T helper type 17 response with up‐regulation of IL‐17 and excessive infiltration of neutrophils, characteristics of psoriatic diseases.^115^ It is not surprising that AIM2, a receptor involved in the recognition of cytosolic DNA, is involved in psoriatic diseases as cytosolic DNA has been shown to be responsible for inflammasome activation in keratinocytes during psoriasis.^116^

Interestingly, a recent study points to a role in AIM2 in immune memory of epithelial cells.^30^ Using epithelial cell stem cells they determined that these cells possess immune memory, enabling a rapid second response after a previous exposure to inflammation. This striking capacity of skin epithelial stem cells is dependent on AIM2 and downstream of IL‐1*β* as *Il‐1β* ^−/−^ and *Il‐1r1* ^*−/−*^ but not *Il‐18* ^*−/−*^ failed to induce immune memory. Given the above‐mentioned role of AIM2 in recurrent skin diseases such as psoriasis, we could speculate that both mechanisms might be linked, suggesting major implications for immune memory of epithelial cells in chronic inflammatory diseases.

Finally, the role of IL‐18 in skin has been widely studied. In fact, IL‐18 has been linked with psoriatic disease, where increased caspase‐1 activity has been reported.^117^ Variations in the IL‐18 gene have also been associated with increased risk of atopic eczema, a chronic inflammatory condition of the skin.^118^

### Final remarks

In this review we have presented the involvement of inflammasome activation in the role of epithelial cells as mediators of the immune response. We have reviewed how epithelial cells are able to release IL‐1*β* and IL‐18. However, despite the relatively low concentration of these cytokines released by epithelial cells compared with macrophages, it is important to mention that in healthy conditions epithelial cells are relatively more abundant in barriers than immune cells. Therefore, the epithelial compartment could contribute greatly to the inflammatory response to pathogens, at least in early infection when not many immune cells have been recruited.

We have highlighted the diversity of responses and of sensor proteins involved in the inflammasome response. The differential expression of sensor molecules and inflammasome components and mediators in the different types of epithelial cells will shape the immune response according to the needs and threats of each specific site. Similarly, the types of pathogens and threats that each barrier will face vary from one epithelium to another, raising the question of whether there is a general mechanism leading to inflammasome activation in epithelial cells or the response is specific to each epithelium and threat. The increasing body of publications in the field points in the direction of several types of inflammasomes involved in different epithelia, making the response tissue‐dependent. We conclude that, in order to better understand this question, the field would benefit from a broad study comparing the involvement of different inflammasomes in the different epithelia. Even more, it is not always clear and differs from one cell line model to another what inflammasome components are present in each epithelium. In our review we have highlighted several examples of differential expression of inflammasome components or pro‐inflammatory cytokines in different cell types or tissues. We could hypothesize that within one organ, different types of epithelial cells will express different inflammasome components, including cytosolic sensors and effector caspases. That would ensure a diverse, adapted and targeted response and prevent a much feared loss of epithelial integrity by massive activation of inflammasomes. It is also important to highlight that most of the available data on the role of inflammasomes in epithelial cell immunity has been obtained using either human cell lines, or primary epithelial cells from mouse. Key differences between human and murine inflammasomes have already been described (for instance NLRC4 is not activated by the same components of bacterial flagellum in mouse and humans and DNA inflammasome activation in human monocytes depends on NLRP3 instead of AIM2^119^, ^120^). Therefore, more studies are needed in human lung epithelial cells to confirm the findings mentioned in this review.

Gasdermin‐D, as mentioned before, is the executor of pyroptosis. However, its role and function in non‐haematopoietic cells is still not well characterized. We could speculate that after inflammasome activation in epithelial cells, the triggering of pyroptosis could have two possible outcomes, both of them highlighting the need for a tight control of pyroptosis in epithelial cells. Pyroptosis during infection could be detrimental (and would be somehow silenced or dampened in epithelial cells) by causing membrane integrity loss. This result is in line with the previously mentioned report showing accumulation of pro‐IL‐1*β* during *P. gingivalis* infection of the oral mucosa. Nevertheless, pyroptosis could also be beneficial during epithelium colonization, as could a mechanism of containing the infection and avoiding further invasion of internal layers of the epithelium. To reinforce this idea, during *Salmonella* infection caspase‐1 and gasdermin‐D cleavage are required for IEC pyroptosis but not for IEC expulsion.^41^ In fact, in that study, the authors demonstrate that during the death and expulsion of IEC, the epithelial integrity is maintained through the rearrangement of neighbouring cells. This result also links with the discovery that small amounts of caspases‐1 are required for gasdermin‐D cleavage (meaning that even a weak activation of inflammasome produces cell death) while detecting active IL‐1*β* requires higher levels of processed caspases‐1.^121^ In such a scenario, weak invasion of the epithelial layer would lead to epithelial cell death and expulsion but still no immune response as the threshold to release IL‐1*β* is much higher than for cell death. This mechanism, potentially occurring with low infection rates where few cells are being infected, could be important to maintain the equilibrium with normal microbiota.

The recent discovery of inflammasome inhibitors such as MCC950 or boron‐based compounds is opening the path to the future treatment of inflammasome‐related pathologies with specific drugs.^122^, ^123^ However, while the field is moving towards better drugs, there is also an increasing body of evidence that inflammasome activity is key in the immune response of many cell types. The potential specific targeting of either immune cells or epithelial cells in different inflammasome‐related pathologies would be beneficial. Such a specificity would allow the targeting of the over‐activation of inflammasome in cells where its activity is detrimental while allowing the normal inflammasome function in other cells.

In conclusion, the role of the inflammasomes in epithelium has been extensively reported. We hypothesize that their contribution to the triggering of inflammation, in opposition to the common belief, is far from being negligible. This combination of findings provides some support for the conceptual premise that targeting inflammasome activity in epithelial cells might be essential for further treatment of a wide range of inflammatory disorders. There is then, abundant room for further progress in elucidating the mechanism of inflammasome activation in epithelial cells and investigating whether it is similar to that in macrophages or not.

## Disclosures

The authors have declared that no conflict of interest exists.
